# WORKING TOWARD A LEARNING COMMUNITY ENABLING LIVING WELL WITH DEMENTIA

**DOI:** 10.1093/geroni/igac059.1424

**Published:** 2022-12-20

**Authors:** Simone de Bruin, Franka Bakker

**Affiliations:** Windesheim University of Applied Sciences, Zwolle, Overijssel, Netherlands; Windesheim University of Applied Sciences, Zwolle, Overijssel, Netherlands

## Abstract

Health and social care professionals play an important role in enabling to live well with dementia. Supporting living well from a psychosocial approach to care requires new ways of working and different competences compared to a medical-somatic approach to care. Interviews and group meetings with future (i.e. students) and current health and social care professionals organized in 2021, however, revealed that training to develop such competences and adopt these new ways of working receive little attention in the curricula of existing training programs. They further experience that their organisations insufficiently facilitate them (e.g. in terms of time, creating a positive learning climate) to actually use these competences and innovate their ways of working. Therefore, there is a need for creating innovative learning environments in the form of learning communities. These collaborations of stakeholders from science, practice, and education aim to accelerate innovation in dementia care based on lifelong learning and development.

